# Association between CAR T-cell persistence and improved response to salvage radiotherapy in patients with DLBCL

**DOI:** 10.2340/1651-226X.2026.46287

**Published:** 2026-09-17

**Authors:** Jiaqi Fan, Nadine Kutsch, Jan-Michel Heger, Philipp Gödel, Eva Heger, Henning Gruell, Philipp Linde, Simone Ferdinandus, Johannes Rosenbrock, Hendrik Dapper, Emmanouil Fokas, Peter Borchmann, Christian Baues

**Affiliations:** a Department of Radiation Oncology, Cyberknife and Radiation Therapy, Faculty of Medicine and University Hospital Cologne, University of Cologne, Cologne, Germany; b Department I of Internal Medicine, Center for Integrated Oncology Aachen Bonn Cologne Düsseldorf, University of Cologne, Medical Faculty and University Hospital Cologne, Cologne, Germany; c Cancer Center Cologne Essen, Partner Site Cologne, Cologne, Germany; d Cologne Lymphoma Working Group (CLWG), Cologne, Germany; e Mildred Scheel School of Oncology Aachen Bonn Cologne Düsseldorf (MSSO ABCD), Cologne, Faculty of Medicine and University Hospital Cologne, Cologne, Germany; f Institute of Virology, Faculty of Medicine and University Hospital Cologne, University of Cologne, Cologne, Germany; g German Center for Infection Research (DZIF), Partner Site Bonn-Cologne, Cologne, Germany; h Department of Radiation Oncology, Marienhospital Herne, Ruhr University Bochum, Bochum, Germany

**Keywords:** lymphoma, large B-cell, diffuse (MeSH ID, D016403), immunotherapy, adoptive (MeSH ID, D016219), CAR T-cell therapy, salvage radiotherapy

## Abstract

**Background and purpose:**

While Chimeric antigen receptor (CAR) T-cell therapy has transformed treatment of relapsed/refractory diffuse large B-cell lymphoma (DLBCL), over half of patients relapse within 1 year with poor prognosis. Salvage radiotherapy (sRT) achieves high local response rates, but which subgroups benefit most and whether CAR T-cell persistence influences sRT response remains unclear.

**Patient/material and methods:**

We retrospectively analyzed DLBCL patients receiving sRT after CAR T-cell therapy at our center. CAR transgene levels were quantified by quantitative polymerase chain reaction (qPCR), and associations between CAR T-cell kinetics at relapse, sRT response, and survival were evaluated.

**Results:**

CAR transgene data were available for 13 patients, (18 lesions treated with sRT). Toxicity was mild (grade ≤ 2). The local response rate was 83% (15/18 lesions). Three CAR transgene kinetic patterns were identified at relapse: increased-CAR (second increase in transgene levels, *n* = 4), persisted-CAR (persistence > 6 months, *n* = 4), and decreased-CAR (decline or absence, *n* = 5). Local response rates were 100% in both the increased- and persisted-CAR groups versus 57% (4/7 lesions) in the decreased-CAR group, 12-month local control rates were 83, 100, and 14%, respectively. Twelve-month overall survival was 100% in both increased-CAR and persisted-CAR groups versus 20% in the decreased-CAR group.

**Interpretation:**

sRT may provide durable local control in relapsed DLBCL after CAR T-cell therapy. Persistent CAR T-cell activity at relapse may help identify patients most likely to benefit from comprehensive sRT. Given the small, heterogeneous cohort, these findings are hypothesis-generating and require validation in larger series.

## Introduction

Chimeric antigen receptor (CAR) T-cell therapy has transformed the management of relapsed or refractory (R/R) B-cell malignancies; however, more than half of treated patients experience disease progression within the first 12 months [1–3], and their prognosis is dismal. Large real-world series consistently report a median overall survival (OS) of only 5–8 months after post-CART progression in R/R B-cell lymphoma [4–6]. This is now a highly studied area, and no standard approach has yet been established for this patient group.

Several retrospective analyses suggest that salvage radiotherapy (sRT) can achieve high in-field response rates (about 80%) and durable local control [7–10]. Patients with localized relapse appear to respond particularly well to sRT, with some achieving durable remission after sRT alone [9, 10]. Whether these favorable outcomes reflect localized disease biology, limited tumor burden, or synergy between sRT and residual CAR T-cell activity remains unclear, as the role of CAR T-cell persistence in durable disease control after sRT has not been investigated.

In this retrospective study, we assessed the efficacy of sRT in patients who relapsed after CAR T-cell therapy and explored whether CAR T-cell persistence was associated with treatment response and clinical outcomes. Our analysis aims to provide an additional aspect for patient selection for sRT and to offer a rationale for further clinical trials investigating the combination of radiotherapy with cellular immunotherapy.

## Patients/material and methods

We conducted a single-center retrospective analysis of patients who received sRT at our institution. Patients were eligible if they had diffuse large B-cell lymphoma (DLBCL), had received CAR T-cell therapy, and subsequently underwent sRT as the first salvage therapy due to post-CART progression or residual disease. All data related to sRT were collected retrospectively.

Staging before and after sRT was generally performed using positron emission tomography/computed tomography (PET/CT), unless limited by poor clinical condition or rapidly progressive symptomatic disease. Treatment response was defined as complete response (CR) or partial response (PR), based on the Lugano criteria [11], or on clinical symptom control in cases where imaging was not available. Localized relapse was defined as disease progression involving no more than two lymph node regions and two extranodal lesions on the same side of the diaphragm [9]. The toxicity of the RT was graded according to Common Terminology Criteria for Adverse Events (CTCAE) [12].

To analyze patient outcomes following salvage therapy, OS was defined as the time from the diagnosis of post-CART progression or residual disease until death from any cause or was censored at the date of last follow-up. Progression-free survival (PFS) was defined as the time from the same starting point until disease progression or death from any cause or was censored at the date of last follow-up.

## Measurement of CAR T-cell persistence

CAR T-cell levels in peripheral blood were quantified by quantitative reverse transcription polymerase chain reaction (qRT-PCR)targeting an HIV-1 derived sequence within the CAR transgene originating from the lentiviral vector used for T cell transduction. In most patients, monitoring was routinely performed on days 4 and 7, at weeks 2 and 4, and at months 3, 6, 9, and 12 after CAR T-cell infusion to track expansion kinetics and CAR T-cell persistence. Additional assessments were obtained at the initiation of any salvage therapy, at the treating physician’s discretion. Patients lacking CAR T-cell persistence data at the time of disease progression were excluded.

Results of the measurements were expressed as the ratio of the lentiviral nucleic acids copy number to the total nucleated cell count in buffy coats isolated from whole blood samples. Lentiviral copies were measured with the cobas 6800 HIV-1 assay, which amplifies the HIV-1 LTR DNA and RNA. The total cell count was determined using a published qPCR protocol for β-globin DNA [13], which is chromosomally present in nucleated cells. Quantification of β-globin was performed using the β-globin standard from the LightCycler Control Kit DNA (Roche), and qPCR was conducted on the LightCycler 480 II system (Roche).

CAR transgene levels were quantified by PCR, providing values relative to the number of nucleated cells. Data on absolute CAR T-cell counts (e.g. by flow cytometry) were not available. We analyzed the CAR persistence at the time of relapse, and the change in CAR transgene levels throughout the disease course. As relative PCR values may not be directly comparable across patients (e.g. due to differences in the number of integrated lentiviral vector genomes and/or leukocyte numbers), no fixed threshold was applied.

The statistical analyses were performed with Python (Version 3.10.2).

## Results

Between August 2018 and May 2023, a total of 21 DLBCL patients received sRT following CAR T-cell therapy. Eight of the 21 patients lacked valid post-relapse CAR transgene measurements and were excluded from this analysis. The lack of measurement was mainly due to a technical limitation: the RT-PCR assay targets an HIV-1 derived sequence specific to the lentiviral vector, which is absent in γ-retrovirally transduced products such as axicabtagene ciloleucel, received by most excluded patients. The few other patients were treated early, when CAR transgene persistence was not yet routinely measured at the time of relapse.

Thirteen patients were included in the analysis. Of these, nine patients were treated with Tisagenlecleucel (tisa-cel), one with Lisocabtagene maraleucel (liso-cel), and three received CAR T-cells as part of a phase II clinical trial. Eight patients showed a therapeutic response, with complete metabolic response (CMR) observed in six patients. The median time from CAR T-cell infusion to the initiation of sRT was 3.6 months (range: 17 days to 23 months).

At the time of disease progression after CAR infusion, 10 patients experienced localized relapses involving 1–2 lesions. Salvage RT was delivered to all metabolically active sites as comprehensive sRT. In contrast, three other patients presented with advanced-stage disease progression and received focal sRT with palliative intent, either for symptom relief (dyspnea or pain) or for tumor debulking. Among the 10 patients with localized relapse, 5 had achieved CMR after CAR T-cell infusion but subsequently developed 1–2 new lesions, whereas the other 5 had never achieved CMR after CAR infusion and showed progression of 1–2 pretherapeutic lesions.

sRT was applied for 18 lesions in 13 patients with a median dose of 40 gray (Gy). sRT were mostly normo-fractionated (13/18) or moderately hypo-fractionated until a single fraction dose of 3 Gy (4/18). One stereotactic sRT was delivered to a lesion in the left lower lobe of the lung, with a total dose of 30 Gy administered in three fractions of 10 Gy each. Sites of RT included: subcutaneous (*n* = 3), axillary (*n* = 1), chest (*n* = 4), abdominal (*n* = 2), retroperitoneal (*n* = 4), pelvic (*n* = 2), and bone (*n* = 2). RTs were well tolerated. Either no toxicity or only mild toxicity (≤ grade 2) had been documented. In patient no. 9, sRT to the abdominal bulk was discontinued after 10 Gy in 5 fractions because of further tumor progression and neurological deterioration. Further patient characteristics at the time of sRT are summarized in Table 1; sRT treatment details are shown in Table 2.

**Table 1:** Patients’ characteristics at the time of salvage radiotherapy, extent of relapse, and salvage treatment strategy.

No.	Age (years)	Best response to CAR T-cell therapy	LDH	SUVmax	Bulky disease	Extent of relapse	Time from CAR T-cell therapy to sRT (months)	Compre-hensive sRT	Salvage therapy
**Decreased-CAR group (*n* = 5)**									
13	69	PD	Normal	11.1	Yes	Advanced stage	3.3	No	Combined
12	81	PMR	465	6.4	No	Localized	2.6	Yes	RT alone
11	72	PD	338	Unknown	Yes	Advanced stage	0.6	No	Combined
10	72	SD	Normal	9.8	No	Localized	3.9	Yes	RT alone
9	50	CMR	1632	Unknown	Yes	Advanced stage	7.2	No	Combined
**Increased-CAR group (*n* = 4)**									
8	30	PD	331	19.9	No	Localized	1.4	Yes	RT alone
7	68	SD	Normal	4.7	No	Localized	3.0	Yes	RT alone
6	33	CMR	Normal	5.1	No	Localized	3.7	Yes	RT alone
5	44	PMR	Normal	6.3	No	Localized	2.1	Yes	RT alone
**Persisted-CAR group (*n* = 4)**									
4	62	CMR	Normal	20.9	No	Localized	7.0	Yes	RT alone
3	58	CMR	Normal	10.5	No	Localized	18.4	Yes	RT alone
2	62	CMR	Normal	6.4	No	Localized	3.6	Yes	RT alone
1	60	CMR	Normal	Unknown	No	Localized	22.6	Yes	Combined

Patient numbering corresponds to Table 2 and Figure 3. Detailed patient characteristics (LDH, SUVmax on PET/CT, bulky disease, and extent of relapse) at the time of sRT. Comprehensive sRT: sRT delivered to all metabolically active lesions; salvage therapy: combined sRT and systemic therapy versus sRT alone. Time from CAR T-cell therapy to sRT is given in months after CAR T-cell infusion. LDH is reported in U/L; ‘Normal’ denotes a value of < 250 U/L. CAR T: CAR T-cell therapy; sRT: salvage radiotherapy; LDH: lactate dehydrogenase; SUVmax: maximum standardized uptake value; CMR: complete metabolic response; SD: stable disease; PD: progressive disease.

**Table 2:** Time intervals from CAR T-cell infusion to relapse, CAR transgene PCR measurements and sRT, sRT treatment details, and outcome after sRT.

No.	Time from infusion to relapse (months)	Time from infusion to CAR measurement 1 (months)	Time from infusion to CAR measurement 2 (months)	Time from infusion to sRT (months)	PCR fold difference (measurement 2 / measurement 1)	Total Dose of sRT (Gy)	Fractions	Total dose, second lesion (Gy)	Fractions, second lesion	Local sRT response	Re-progression	PFS (months)
**Decreased-CAR group (*n* = 5)**												
13	2.3	n/a	3.0	3.3	n/a (undetectable)	36	12	-	-	PR	Yes	22.1
12	1.0	0.5	1.2	2.6	0.045	40	20	40	20	PR	Yes	4.1
11	0.5	0.4	0.5	0.6	0.251	40	19	-	-	PR	Yes	2.7
10	2.8	0.5	5.4	3.9	0.002	40	20	40	20	PD	Yes	2.4
9	7.0	n/a	5.4	7.2	n/a (undetectable)	10*	5	-	-	PD	Yes	0.4
**Increased-CAR group (*n* = 4)**												
8	0.9	0.5	1.1	1.4	10.422	30	3	45	18	PR	No	40.8
7	1.0	0.4	1.2	3.0	1.340	36	12	-	-	PR	No	35.0
6	2.4	0.5	2.4	3.7	4.908	39.6	22	39.6	22	CR	No	26.6
5	1.1	1.8	3.4	2.1	3.042	40	20	-	-	PR	Yes	5.1
**Persisted-CAR group (*n* = 4)**												
4	6.2	n/a	6.2	7.0	n/a	40	20	-	-	CR	No	43.9
3	16.8	n/a	17.1	18.4	n/a	39	13	-	-	CR	No	27.9
2	3.0	n/a	10.8	3.6	n/a	40	20	-	-	CR	No	21.4
1	21.2	n/a	36.3	22.6	n/a	46	23	46	23	CR	Yes	17.7

Patient numbering corresponds to Table 1 and Figure 3. All time intervals are counted from the date of CAR T-cell infusion (day 0) to the date of the respective event and are reported in months (1 month = 30.44 days). A value of 3.3 in the column ‘Time from infusion to sRT’ therefore means that salvage radiotherapy started 3.3 months after CAR T-cell infusion. Time from infusion to relapse refers to the date of relapse diagnosis (imaging-confirmed progression or metabolically active residual disease). CAR measurement 1 and CAR measurement 2 are the CAR transgene PCR samples obtained closest before and after the date of relapse diagnosis, respectively; ‘n/a’ indicates that no CAR measurement 1 was available for that patient. PCR fold difference = CAR measurement 2 divided by CAR measurement 1; the reproducibility of qPCR copy numbers typically lies within a range of approximately two-fold; ‘n/a’ indicates that a fold difference could not be calculated because CAR measurement 1 was not available; ‘n/a (undetectable)’ indicates that CAR transgene was not detectable at the available measurement. Local sRT response was assessed on the first post-sRT imaging within the irradiated volume. Re-progression denotes any subsequent disease progression after sRT. PFS is measured from the time from the diagnosis of post-CART progression or residual disease until disease progression or death from any cause or was censored at the date of last follow-up, in months. sRT: salvage radiotherapy; PCR: polymerase chain reaction; Gy: Gray; PFS: progression-free survival; CR: complete response; PR: partial response; PD: progressive disease.

* In patient no. 9, sRT to the abdominal bulk was discontinued after 10 Gy in 5 fractions because of further tumor progression and deterioration in general condition.

**Figure 1:**
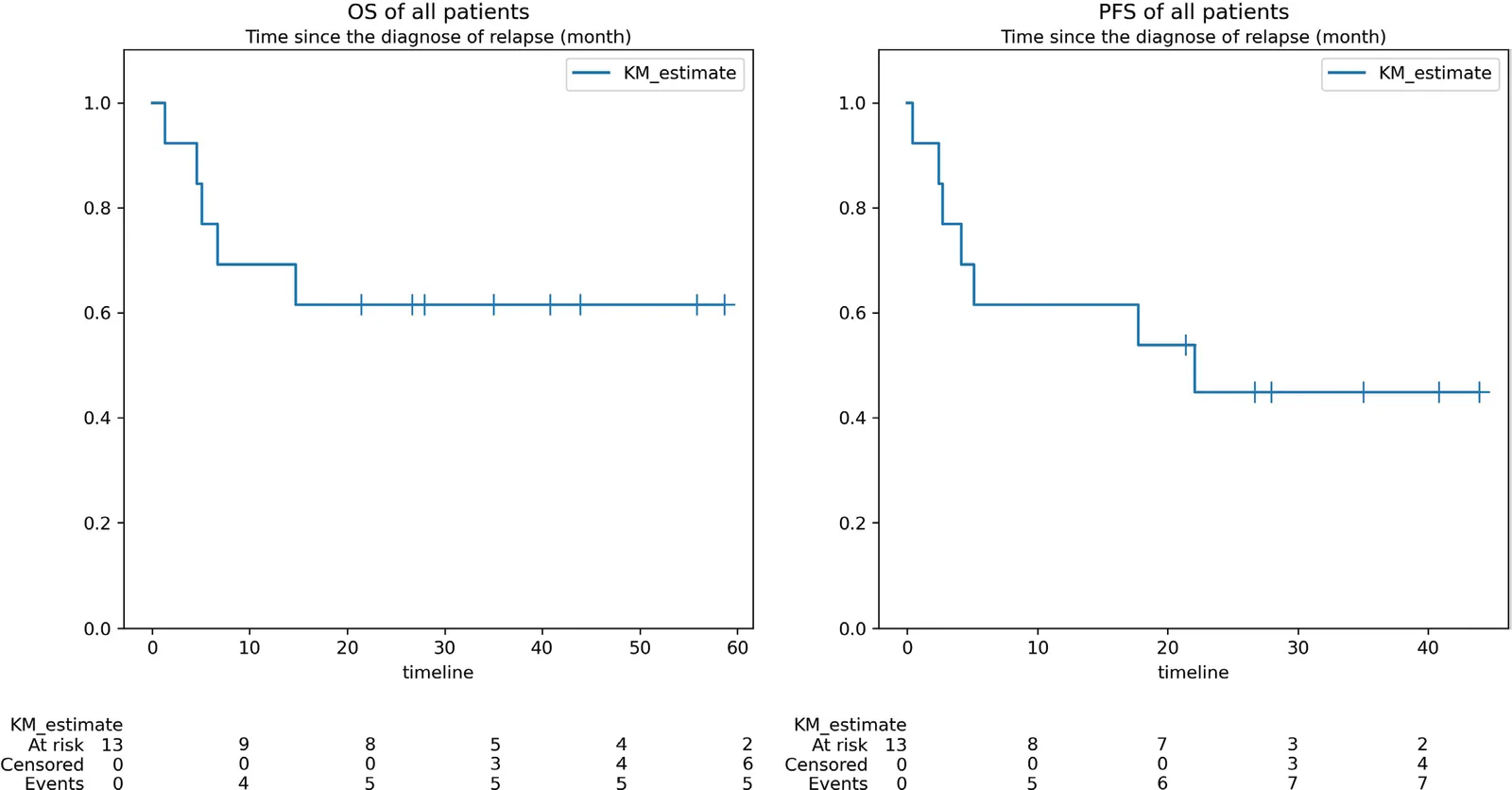
Kaplan–Meier estimates of OS and progression-free survival in all 13 patients (months). Left: OS from the diagnosis of post-CAR-T progression or residual disease; right: progression-free survival from the diagnosis of post-CAR-T progression or residual disease. CAR T: CAR T-cell therapy; PFS: progression-free survival; OS: overall survival.

The local response rate to all sRT treatments was 83% (15/18 lesions), with a median duration of local control of 10.7 months. After the diagnosis of progression, the 1-year OS rate for all patients was 69.2%, and the 1-year PFS rate was 61.5%. The OS and PFS of all patients are shown in Figure 1.

## CAR transgene level at the time of relapse

**Figure 2:**
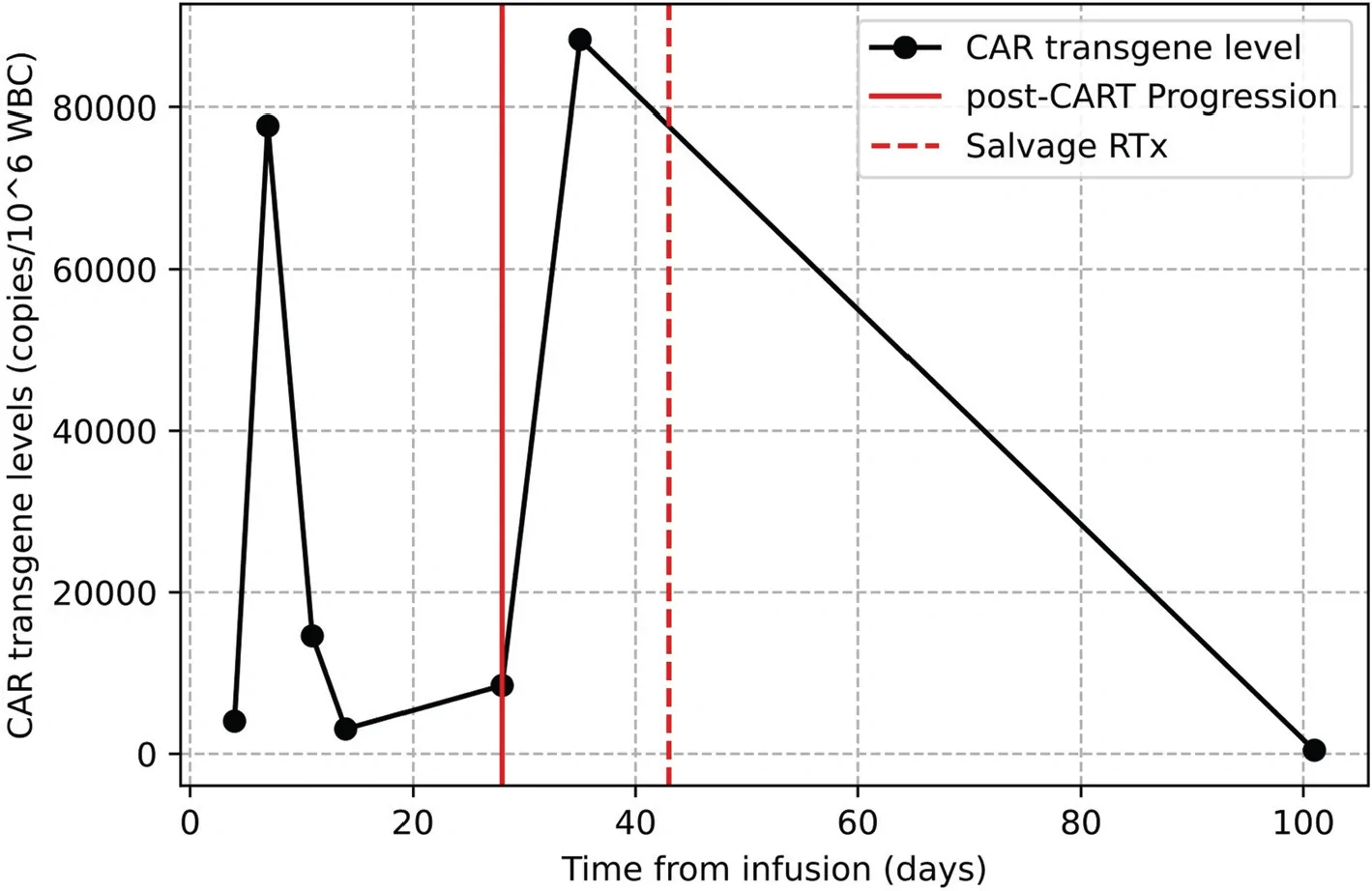
The kinetics of the CAR transgene level in one patient from the increased-CAR group, started from the day of CAR T-cell infusion. The time of post-CAR-T progression is marked with a red solid line, and the day of salvage RTx with a red dashed line. Two months after salvage RTx, the patient underwent allogeneic stem cell transplantation. Thereafter, CAR T-cells were no longer detectable (after day 100). CAR T: CAR T-cell therapy; RT: radiotherapy.

Interestingly, we observed a second increase in CAR transgene levels at the time of relapse in four patients, prior to any salvage therapy. All of them had initially demonstrated peak expansion after CAR infusion, followed by a subsequent decline. At relapse (28–74 days after infusion), CAR transgene levels rose again compared to the last measurement prior to relapse, with increases ranging from 1.3- to 10.4-fold. The transgene level kinetic of one of these four patients are shown in Figure 2.

In contrast, two other patients showed no detectable CAR persistence at the time of relapse. Three additional patients exhibited peak expansion after CAR infusion, followed by a decline in transgene levels, which continued to decrease further after relapse.

The remaining four patients all achieved CMR after CAR T-cell infusion and demonstrated long-term CAR persistence (> 6 months), documented at the time of localized relapse (1–2 lesions) or thereafter. Three of these patients experienced late relapse (6–21 months post-infusion). As PCR testing for CAR transgene levels was performed more frequently during the first 3 months post-infusion, pre-relapse measurements were not available for those four patients, and CAR T-cell dynamics at relapse could not be assessed in these patients. Notably, one of the four received sRT followed by a second CAR T-cell infusion 2 months later.

Based on these distinct patterns in CAR transgene kinetics, patients were stratified descriptively into three groups, defined either by the CAR transgene level after disease progression or by the within-patient direction of change between measurements obtained around the time of relapse: the increased-CAR group (*n* = 4), with repeatedly rising levels (1.3–10.4-fold); the decreased-CAR group (*n* = 5), with declining (0.25–0.002-fold) or undetectable levels; and the persisted-CAR group (*n* = 4), with long-term CAR persistence (> 6 months) but unknown trajectory at the time of relapse. All patients in the persisted-CAR group had a localized relapse after a CMR to CAR T-cell therapy; for this group, no valid preceding measurement was available for comparison. Patient 7, in whom the smallest change was observed (1.3-fold), was assigned to the increased-CAR group on the basis of the direction rather than the magnitude of change. The timing of measurements relative to relapse diagnosis and initiation of sRT, together with the extent of change where available, is provided in Table 2.

Patients in the decreased-CAR group more often presented with advanced-stage disease (60% vs. 0 and 0%) and bulky disease (60% vs. 0 and 0%) and were older (median age 72 vs. 39 and 61) compared with those in the increased-CAR and persisted-CAR groups. Detailed patient characteristics are summarized in Table 1.

**Figure 3:**
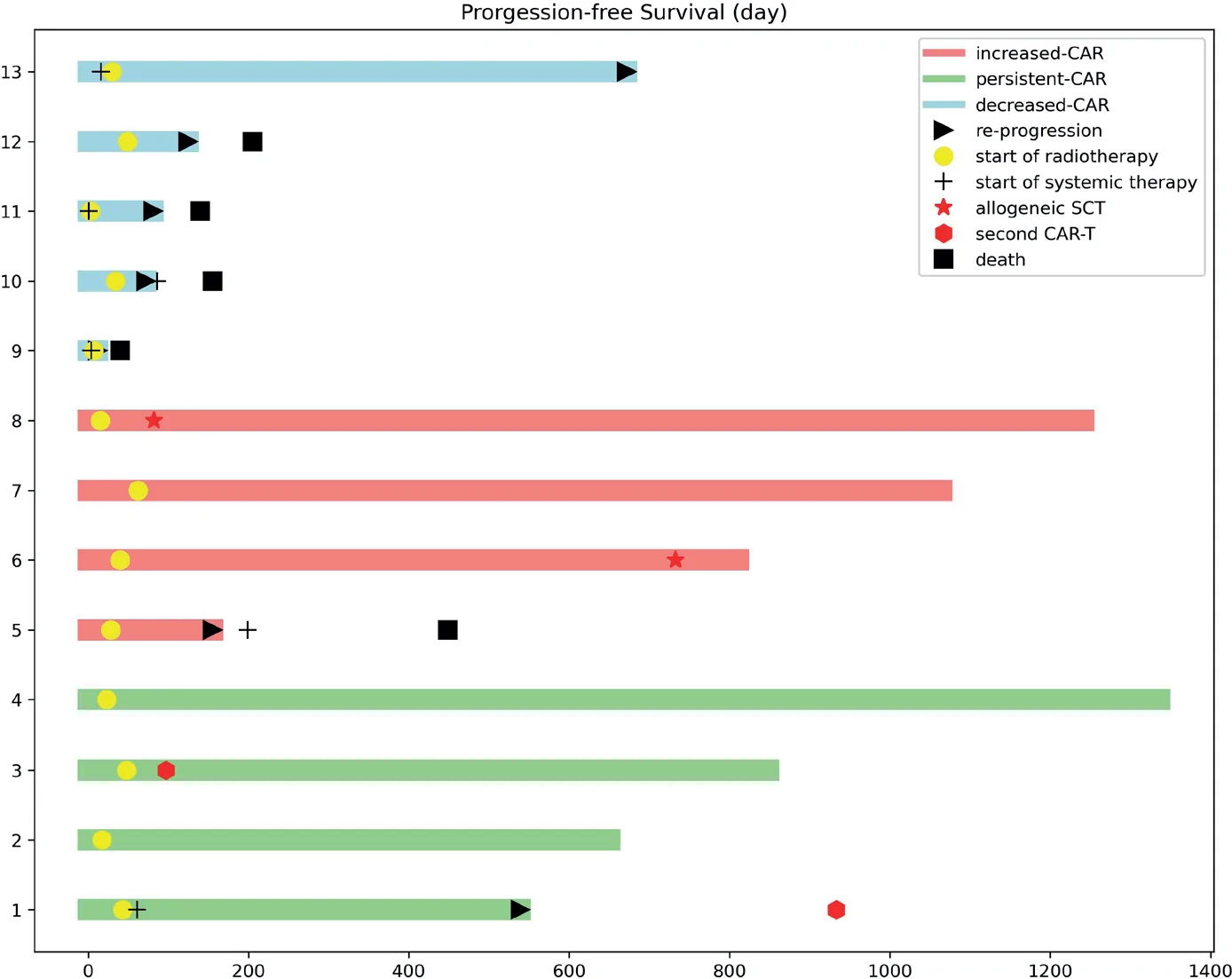
Swimmer plot illustrating individual patient clinical courses and treatment outcomes for all 13 patients from the diagnosis of post-CAR-T progression until subsequent disease re-progression or last follow-up. Each horizontal bar represents one patient, and the bar length indicates the duration of progression-free survival (day). Patient numbering corresponds to Tables 1 and 2. Patients 1–4 (green) belong to the persistent-CAR group, patients 5–8 (red) to the increased-CAR group, and patients 9–13 (blue) to the decreased-CAR group. Symbols indicate clinical events, including salvage radiotherapy (start of radiotherapy: yellow circle), initiation of systemic therapy (start of systemic therapy: cross), allogeneic stem cell transplantation (allogeneic SCT: red star), second CAR T-cell therapy (second CAR-T: red circle), re-progression (arrow), and death (square). CAR-T, CAR T-cell therapy.

In the increased-CAR group, all patients received sRT alone. Two young patients in good general condition subsequently underwent allogeneic stem cell transplantation, 2 and 22 months after sRT, in both cases without preceding progression. In the persisted-CAR group, one patient received combined salvage therapy (sRT plus systemic therapy), and another received a repeat CAR T-cell infusion 20 months after the first, 2 months after sRT and without preceding progression, because CAR transgene levels were considered low relative to our institutional experience. In the decreased-CAR group, three patients received combined salvage therapy, as sRT could not be directed to all metabolically active lesions; the remaining two received sRT alone until further progression. The clinical course and treatment outcomes of all 13 patients are shown in Figure 3.

We observed better treatment response, longer response duration, and improved PFS in patients of the increased-CAR group and persisted-CAR group, compared with patients of the decreased-CAR group.

Local response rates and the duration of local control were higher in the increased-CAR and persisted-CAR groups, with local responses observed in 100% of lesions in both groups (6/6 and 5/5 lesions, respectively), compared with 57% (4/7 lesions) in the decreased-CAR group. At 12 months after relapse, seven of eight patients in the increased- and persisted-CAR groups remained free from local progression, whereas only one of five patients in the decreased-CAR group was alive with maintained local control. The corresponding 12-month local control rates were 83% (5/6 lesions) and 100% (5/5 lesions), respectively, compared with 14% (1/7 lesions) in the decreased-CAR group.

**Figure 4:**
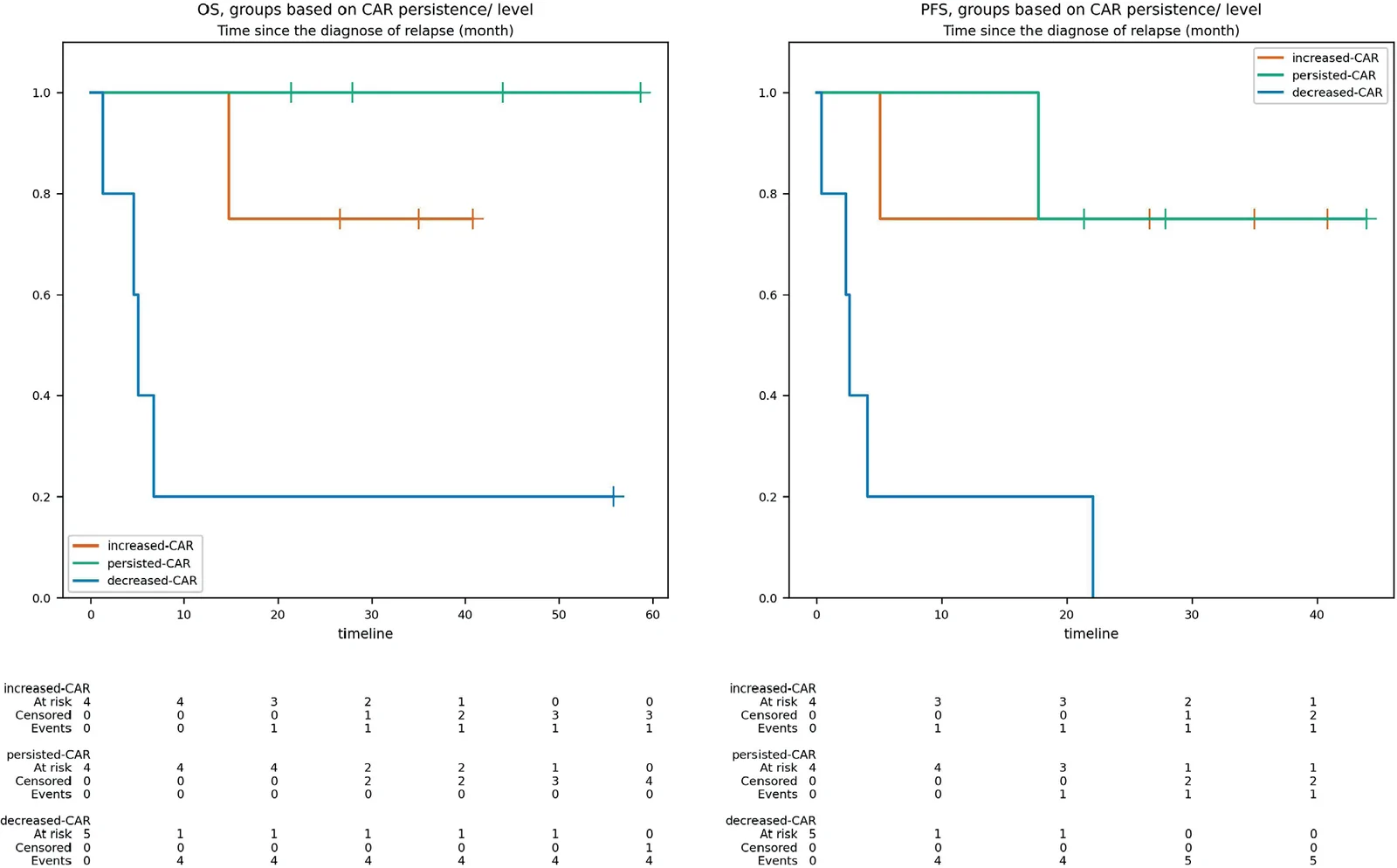
Kaplan–Meier estimates of OS (left) and PFS (right) from the diagnosis of post-CAR T-cell progression or residual disease, by CAR transgene kinetics at relapse: increased-CAR (n = 4), persisted-CAR (n = 4), and decreased-CAR (n = 5). Vertical marks indicate censored observations. CAR T: CAR T-cell therapy; OS: overall survival; PFS: progression-free survival.

Survival outcomes also favored the increased-CAR and persisted-CAR groups. At 12 months post-relapse, all patients in the increased-CAR and persisted-CAR groups were alive, with only one progression in the increased-CAR group. The 12-month OS rate was 100% in both groups, and the 12-month PFS rates were 75 and 100%, respectively. By contrast, four of five patients in the decreased-CAR group experienced progression and died within 6 months, resulting in a 12-month OS rate of 20% and a PFS rate of 20%. The only long-term survivor in this group (patient 13, Figure 3) received lenalidomide and rituximab plus sRT to inguinal lymph nodes, followed 2 years later by sRT to a progressive pulmonary lesion, achieving a CR lasting > 2 additional years. OS and progression-free survival for all three groups are shown in Figure 4.

## Discussion and conclusion

In the context of CAR T-cell therapy for r/r DLBCL, radiotherapy has demonstrated an important role as both bridging and salvage therapy in several previous retrospective studies, achieving high local response rates with limited toxicity [7,9, 14–16]. To our knowledge, this is the first study to investigate the impact of CAR T-cell persistence on the response to sRT in patients with disease progression after CAR T-cell infusion.

A few previous studies showed evidence that patients with localized relapse benefit more from sRT. The first report on sRT from Imber et al. in 2020 and the data from another multicenter study both demonstrated that patients with localized relapses appeared to have better local responses to sRT and improved post-sRT outcomes compared to those with advanced-stage relapses [7, 9]. A further retrospective study by Saifi et al. reported the potential benefit of consolidative radiotherapy for residual fluorodeoxyglucose (FDG) uptake on day +30 post-CART in B-cell non-Hodgkin lymphoma (NHL), with the most pronounced effect observed in patients with ≤ 2 residual FDG-avid sites [8]. These studies also reported the potential for long-term local control following sRT, and potential for improved OS in patients relapsed with limited disease who received comprehensive irradiation of all relapse lesions.

The mechanisms underlying the observation that patients with localized relapse derive greater benefit from sRT remain incompletely understood. We hypothesized that, in this setting, CAR T-cells may retain systemic activity but be limited by local resistance mechanisms. sRT could enhance antitumor effects and thereby overcome this local resistance. Therefore, we analyzed CAR transgene persistence at the time of relapse.

Overall, we observed superior sRT responses and local control in the increased- and persisted-CAR groups compared with the decreased-CAR group, with response rates of 100, 100, and 57%, and corresponding 12-month local control rates of 83, 100, and 14%, respectively. Most patients in the increased- and persisted-CAR groups subsequently achieved long-term remission (7/8), including four who received sRT without additional concomitant or sequential systemic therapy. These findings suggest that durable local control and long-term remission after comprehensive sRT for localized relapse may depend, at least in part, on the persistence of systemically active CAR T-cells.

Two possible models may explain the observed association. In an additive model, persistent CAR T-cells maintain systemic disease control, whereas relapse represents a localized sanctuary or immune escape site where CAR T-cell activity is insufficient. sRT eradicates this site, and the two treatment modalities act without direct biological interaction. In an interactive model, radiotherapy additionally modulates the irradiated tumor microenvironment and enhances local CAR T-cell function, resulting in a synergistic antitumor effect.

This second model is supported by preclinical studies showing synergistic antitumor interactions between RT and CAR T-cells. Irradiation was shown to enhance local CAR T-cell expansion within the tumor microenvironment, induce pro-apoptotic signaling, and reduce immunosuppressive cytokines [17–21]. In B-cell ALL and DLBCL models, low-dose irradiation improved tumor control through activation of the death receptor pathway, a key regulator of CAR T-cell resistance [20, 22]. Likewise, Murty et al. showed that combining whole-body irradiation (WBI) with CAR T-cell therapy produced stronger antitumor responses, longer survival, and durable immune memory upon tumor re-challenge [19]. Remarkably, mice that received the combination therapy were also protected against tumor re-challenge 10 weeks later, simulating a relapse, suggesting the induction of durable immunologic memory and long-term tumor control. Taken together, these findings provide a biological rationale for using sRT as a potential local booster for CAR-T cells.

In our cohort, CAR transgene levels were measured mostly before the start of sRT, as these assays served treatment planning rather than systematic monitoring, and no post-sRT kinetics were obtained. Our study therefore cannot provide direct evidence that radiotherapy itself enhanced CAR T-cell expansion in vivo.

Nonetheless, we consider a hypothesis-generating statement on a possible interaction between sRT and CAR T-cell activity justified. Durable disease control was clearly more frequent in the increased- and persisted-CAR groups than in patients with declining or undetectable CAR transgene levels. All patients had been heavily pretreated, and four of the patients with durable control more than 12 months were treated with sRT without any further salvage systemic therapy. In this relapsed/refractory setting, radiotherapy alone would not readily be expected to produce local disease control of this duration. We therefore consider it biologically plausible that sRT synergized with CAR T-cell mediated tumor control in a subset of patients, in line with the preclinical mechanisms outlined above. CAR T-cell persistence was not assessed in the cohorts reported by Imber et al. and Saifi et al., the durable responses after sRT described there may likewise have occurred in patients with ongoing CAR T-cell activity [7, 8].

Further studies with serial pre- and post-sRT quantification of CAR transgene levels, ideally complemented by absolute CAR T-cell counts and functional characterization, are needed to establish whether sRT and CAR T-cell activity interact biologically.

In addition, several alternative explanations for our findings should be considered, given the small sample size and the imbalance in baseline disease characteristics between the groups. Patients in the decreased-CAR group more frequently had advanced-stage and bulky disease. The better outcomes observed in the increased- and persisted-CAR groups may therefore partly reflect more favorable disease biology, and CAR T-cell persistence may represent a favorable prognostic marker rather than an independent predictor of benefit from sRT.

A previous retrospective study showed that CAR T-cell persistence at 6 months was associated with fewer relapses and longer PFS [23]. An association between CAR T-cell kinetics and treatment response was also reported in translational analyses of ZUMA-1, in which durable responses were associated with CAR T-cell expansion relative to pretreatment tumor burden, product T-cell fitness, and host systemic inflammation [24]. CAR T-cell kinetics may thus be influenced by disease burden as well as by underlying host and product characteristics.

In our study, the better response, local control, and survival observed increased- and persisted-CAR groups may have resulted from residual CAR T-cell activity and its potential interaction with sRT, from more favorable disease biology, or from a combination of both. Because of the limited sample size, we cannot determine whether CAR T-cell persistence predicts benefit from sRT or merely reflects a favorable prognosis.

Furthermore, the initial response to CAR T-cell therapy represents a further imbalance between the groups and may have influenced patient outcomes. All patients in the persisted-CAR group had achieved a CR and subsequently experienced a localized relapse, whereas patients in the increased-CAR and decreased-CAR groups more frequently had a PR or no response. Response to CAR T-cell therapy is itself an established prognostic factor [25], and this imbalance may therefore have contributed to the favorable outcomes observed in the persisted-CAR group. It could not, however, explain the favorable outcomes in the increased-CAR group, whose initial response profile was comparable to that of the decreased-CAR group.

As noted above, the key limitations of our study include its retrospective design, the small sample size, and the heterogeneity of the patient population. As our qPCR-based transgene quantification method measures CAR T-cell levels relative to all nucleated cells in the analyzed sample, the results may be influenced by the total cell count and the number of integrated vector genomes. All findings are based on clinical observations. We acknowledge that, given the limited number of patients and the absence of a standardized, absolute quantification method, this categorization of CAR transgene kinetics is descriptive and hypothesis-generating rather than a validated classification.

## Conclusion

Our findings suggest that sRT may provide durable local control in patients with relapsed DLBCL after CAR T-cell therapy, particularly in those with evidence of persistent CAR T-cell activity, such as localized relapse after long-term remission or a second increase in CAR levels at the time of relapse. CAR T-cell persistence at relapse may therefore help identify patients most likely to derive durable benefit from comprehensive sRT. Whether the observed association reflects a biological interaction between radiotherapy and CAR T-cells, an additive effect of two treatment modalities acting on distinct disease compartments, or simply more favorable underlying disease biology cannot be determined from our data. Further studies in larger cohorts, including serial pre- and post-sRT quantification of CAR transgene levels and complementary assessments such as flow cytometry, are needed to validate these findings and to better define the optimal integration of sRT into treatment algorithms following CAR T-cell therapy.

## Data Availability

Research data are stored in an institutional repository and will be shared upon request to the corresponding author.
